# Health Professions Digital Education on Antibiotic Management: Systematic Review and Meta-Analysis by the Digital Health Education Collaboration

**DOI:** 10.2196/14984

**Published:** 2019-09-12

**Authors:** Bhone Myint Kyaw, Lorainne Tudor Car, Louise Sandra van Galen, Michiel A van Agtmael, Céire E Costelloe, Onyema Ajuebor, James Campbell, Josip Car

**Affiliations:** 1 Centre for Population Health Sciences Lee Kong Chian School of Medicine Nanyang Technological University Singapore Singapore; 2 Family Medicine and Primary Care Lee Kong Chian School of Medicine Nanyang Technological University Singapore Singapore; 3 Department of Primary Care and Public Health School of Public Health Imperial College London London United Kingdom; 4 Department of Internal Medicine Amsterdam UMC Vrije Universiteit Amsterdam Amsterdam Netherlands; 5 Research & Expertise Center In Pharmacotherapy Education Amsterdam Netherlands; 6 Health Workforce Department World Health Organization Geneva Switzerland

**Keywords:** digital education, antibiotic management, systematic review, meta-analysis, randomized controlled trial

## Abstract

**Background:**

Inappropriate antibiotic prescription is one of the key contributors to antibiotic resistance, which is managed with a range of interventions including education.

**Objective:**

We aimed to summarize evidence on the effectiveness of digital education of antibiotic management compared to traditional education for improving health care professionals’ knowledge, skills, attitudes, and clinical practice.

**Methods:**

Seven electronic databases and two trial registries were searched for randomized controlled trials (RCTs) and cluster RCTs published between January 1, 1990, and September 20, 2018. There were no language restrictions. We also searched the International Clinical Trials Registry Platform Search Portal and metaRegister of Controlled Trials to identify unpublished trials and checked the reference lists of included studies and relevant systematic reviews for study eligibility. We followed Cochrane methods to select studies, extract data, and appraise and synthesize eligible studies. We used random-effect models for the pooled analysis and assessed statistical heterogeneity by visual inspection of a forest plot and calculation of the I^2^ statistic.

**Results:**

Six cluster RCTs and two RCTs with 655 primary care practices, 1392 primary care physicians, and 485,632 patients were included. The interventions included personal digital assistants; short text messages; online digital education including emails and websites; and online blended education, which used a combination of online digital education and traditional education materials. The control groups received traditional education. Six studies assessed postintervention change in clinical practice. The majority of the studies (4/6) reported greater reduction in antibiotic prescription or dispensing rate with digital education than with traditional education. Two studies showed significant differences in postintervention knowledge scores in favor of mobile education over traditional education (standardized mean difference=1.09, 95% CI 0.90-1.28; I^2^=0%; large effect size; 491 participants [2 studies]). The findings for health care professionals’ attitudes and patient-related outcomes were mixed or inconclusive. Three studies found digital education to be more cost-effective than traditional education. None of the included studies reported on skills, satisfaction, or potential adverse effects.

**Conclusions:**

Findings from studies deploying mobile or online modalities of digital education on antibiotic management were complementary and found to be more cost-effective than traditional education in improving clinical practice and postintervention knowledge, particularly in postregistration settings. There is a lack of evidence on the effectiveness of other digital education modalities such as virtual reality or serious games. Future studies should also include health care professionals working in settings other than primary care and low- and middle-income countries.

**Clinical Trial:**

PROSPERO CRD42018109742; https://www.crd.york.ac.uk/prospero/display_record.php?RecordID=109742

## Introduction

Antibiotic resistance is one of the most important public health concerns globally, and the magnitude of the problem is increasing [1]. The World Health Organization (WHO) warns that “without urgent action, we are headed for a post-antibiotic era, in which common infections and minor injuries can once again kill” [2]. Each year, in the United States, at least 2 million people become infected with bacteria that are resistant to antibiotics, and annually, 23,000 people die as a direct result of these infections [1,3]. Indirectly, antibiotic resistance contributes to the increasing costs of health care worldwide. Failure to address the issue of antibiotic resistance is estimated to result in excess of 10 million deaths worldwide and cost up to US $100 trillion annually by 2050 [4].

The main causes of antibiotic resistance include overprescription of antibiotics, overuse of antibiotics in livestock and fish farming, poor infection control in hospitals and clinics, patients not finishing their treatment, a lack of hygiene, and poor sanitation [5-7]. Adding to the problem is a shortage of health manpower and training facilities coupled with a lack of effective educational programs for health care professionals [8]. In line with the aforementioned challenges, the WHO supports countries through its competency framework on antimicrobial resistance (AMR) by providing guidance on the requisite competencies for health workforce learners at preregistration and postregistration levels in order to help address AMR in policy and practice settings [9].

The use of information and communication technology in education, ie, digital education, could improve antibiotic management education [10]. Digital education offers several potential advantages over traditional education methods, such as easier access to learning materials and facilitating the learning process without time or location constraints. Through its range of different modalities, digital education enables different educational experiences with varying forms and levels of interactivity, immersion, duration of the intervention, feedback, etc. Digital education also allows for uncomplicated and scalable dissemination of the latest evidence. For antimicrobial resistance, digital platforms are increasingly being used to deliver education and training, and there is potential to increase their uptake by, for example, ensuring that such programs are accredited and health care professionals are incentivized through various means [11,12]. As antibiotic resistance is the most prevalent form of antimicrobial resistance [13] and can potentially be prevented through health professions education [14,15], this paper focuses on the use different forms of digital education for antibiotic management by pre- and postregistration health care professionals.

Digital education (also known as electronic learning or digital learning) is the act of teaching and learning by means of digital technologies. It is an overarching term for an evolving multitude of educational approaches, concepts, methods, and technologies. Digital education can be further characterized by specific pedagogies and instructional methods, contexts of provision, and technical affordances of hardware and software [16]. It includes, but is not limited to, offline and online digital education [17-19], serious gaming and gamification [20], massive open online courses, virtual reality [21], virtual patient simulations [22], and mobile digital education [23] (Multimedia Appendix 1) [24-28].

Past reviews [29,30] on the effectiveness of educational interventions for the management of antibiotics focused mainly on traditional modes of intervention such as the use of text-based or paper-based interventions. To our knowledge, there are no reviews assessing the effectiveness of digital education for health professions on antibiotic management. To address this gap, we performed a systematic review on the effectiveness of digital technology either alone (single modality), combined with other digital technology (multimodalities), or combined with traditional education (blended digital education) to deliver education on antibiotic management for both pre- and postregistration health care professionals.

## Methods

### Search Strategy and Selection Criteria

We carried out a systematic review and meta-analysis by following Preferred Reporting Items for Systematic reviews and Meta-Analysis guidelines [31]. The protocol for this review was registered in the International Prospective Register of Systematic Reviews (PROSPERO, CRD42018109742) [32]. Randomized controlled trials (RCTs) and cluster RCTs (cRCTs) of pre- and postregistration health care professionals using any type of digital education (either standalone or blended) with any type of control (including traditional education and other forms of digital education) measuring change in clinical practice, knowledge, skills, attitudes, satisfaction, patient-centered outcomes (as primary outcomes); adverse-effects; or economic outcomes (as secondary outcomes) were eligible for inclusion in this review. We included participants and holders of the qualifications listed in the Health Field of Education and Training (section 091) of the International Standard Classification of Education: Fields of Education and Training (United Nations Educational, Scientific and Cultural Organization) and excluded studies with participants from the field of traditional, alternative, and complementary medicine. We excluded crossover trials due to the high likelihood of carry-over effect. A detailed description of the methodology is provided in a previous study by Car et al [16].

We developed a comprehensive search strategy for Medline (Ovid), Embase (Ovid), Cochrane Central Register of Controlled Trials (CENTRAL, Wiley), PsycINFO (Ebsco), Educational Research Information Centre (Ebsco), Cumulative Index to Nursing and Allied Health Literature (Ebsco), and Web of Science Core Collection (Thomson Reuters; see Multimedia Appendix 2 for the Medline [Ovid] search strategy). Databases were searched from January 1, 1990, to September 20, 2018. We chose 1990 as the starting year for our search, as the use of computers was limited to basic tasks prior to this period [16]. We also searched the International Clinical Trials Registry Platform Search Portal and metaRegister of Controlled Trials to identify unpublished trials (see Multimedia Appendix 3 for the lists of Clinical Trial Registries).

The search results from each source were combined in a single library, and duplicate records were removed using reference management software [33]. Three reviewers (BK, GD, and LG) independently screened titles and abstracts of all records to identify potentially relevant studies. We retrieved full-text copies of articles deemed potentially relevant and independently assessed the retrieved articles against the eligibility criteria. Finally, we searched reference lists of all the studies that we deemed eligible for inclusion in our review and relevant systematic reviews.

Three reviewers (BK, GD, and LG) independently extracted relevant characteristics related to participants, intervention, comparators, outcome measures, and results from all the included studies using a standard data collection form. Any disagreements were resolved by consensus through discussion.

### Data Analysis

For continuous outcomes, we reported mean values and SDs of the outcomes in each intervention group along with the number of participants and *P* values. For dichotomous outcomes, we reported risk ratio (RR) with 95% CIs. We were unable to identify a clinically meaningful interpretation of effect size in the literature for digital education interventions. Therefore, in line with other studies in the field, we presented outcomes using postintervention standardized mean difference (SMD) and interpreted the effect size using the Cohen rule [34,35].

Where studies reported more than one measure for each outcome, the primary measure, as defined by the study authors, was used in the analysis. Where no primary measure was reported, the measure that was most consistent with the outcomes presented in other included studies and the first measurement after the intervention were reported.

For cRCTs, we attempted to obtain data at the individual level. In cases where the statistical analysis of the cRCT had already been adjusted for data clustering, we extracted the reported effect estimates and used them directly for our analyses. If a meta-analysis of the included studies was indicated, we assessed statistical heterogeneity by visual inspection of a forest plot and by examining the I^2^ statistic, with I^2^<25%, 25%-75%, and >75% representing a low, moderate, and high degree of heterogeneity, respectively [35]. We used a narrative approach to data synthesis instead of reporting pooled results from meta-analysis if we detected substantial clinical, methodological, or statistical heterogeneity across included studies.

For meta-analysis, we used a random-effects model. Continuous outcome data were presented in the form of standardized mean difference (SMD) (due to the use of different scales), along with 95% CIs. In the analysis of continuous outcomes, we used the inverse variance method. We displayed the result of the meta-analysis in a forest plot that provided effect estimates and 95% CIs for each individual study as well as a pooled effect estimate and 95% CI. We performed meta-analysis using Review Manager Software 5.3 [36]. We adhered to the statistical guidelines described by Higgins et al [35].

Three reviewers (BK, GD, and LG) independently assessed the methodological risk of bias of included studies in accordance with the Cochrane methodology [35]. The following individual elements of RCTs were assessed: random sequence generation, allocation concealment, blinding (outcome assessment), completeness of outcome data (attrition bias), selective outcome reporting (relevant outcomes reported), and other sources of bias (baseline imbalances). For cRCTs, we assessed the risk of the following additional domains: recruitment bias, baseline imbalance, loss of clusters, incorrect analysis, and comparability with individually randomized trials as recommended by Puffer et al [37]. Judgments concerning risk of bias for each study were scored as high, low, or unclear. Studies were judged to be at high risk of bias if there was a high risk of bias for one or more domains or unclear risk of bias in three or more domains. Studies were judged to be at unclear risk of bias if there was an unclear risk of bias for two domains. We incorporated the results of the risk of bias assessment into the review using risk of bias tables, a graph, and a narrative summary.

**Figure 1:**
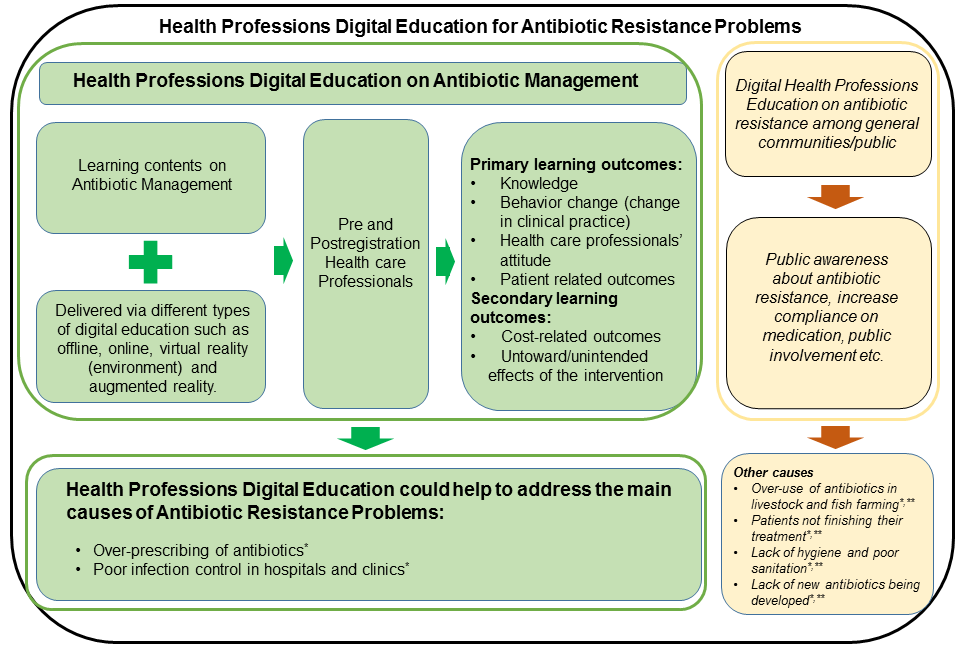
A simplified conceptual framework to address antibiotic resistance by empowering health professions education through digital education. *Causes of antibiotic resistance as per the World Health Organization (2018) data [2]. **Knight Gwenan et al (2018) [38].

We developed a conceptual framework on how different digital education interventions focused on antibiotic management could contribute to an improvement in antibiotic-prescribing practices and a reduction in antibiotic resistance (Figure 1). The aim of the framework was to enable an improvement of health care professionals’ knowledge and skills (as well as other outcomes including patient-related outcomes) by empowering health professions education via digital education. We hope that by facilitating the delivery methods in health professions education, learning outcomes will be improved.

## Results

### Overview

**Figure 2:**
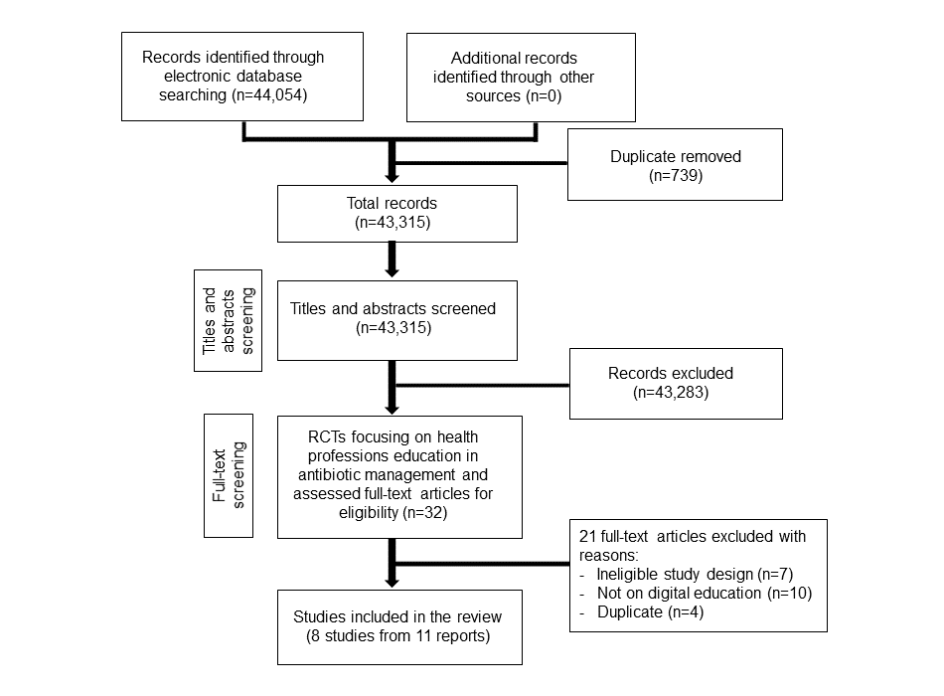
Study selection process. RCT: randomized controlled trial.

Our search strategy retrieved 44,054 unique references (Figure 2). We included 8 studies from 11 reports involving 655 health care practices, 1392 primary care physicians, and 485,632 patients [39-49]. Six studies were cRCTs [41-45,47], and two were RCTs [48,49]. Six studies randomized the participants into two groups, and two studies randomized participants into four groups. All participants were postregistration primary care physicians. The number of participants across the studies ranged from 12 [49] to 479 [47]. Two studies provided additional information for patients and caregivers in the form of leaflets or booklets as a part of the intervention [43,45]. Dekker et al [45] assessed the effects of an intervention that consisted of online digital education for primary care physicians and an information booklet for patients or caregivers. Little et al [43] delivered online digital education targeting primary care physicians and provided interactive booklets as additional resources for physicians to use during consultations with patients.

The content of the interventions focused on different aspects of antibiotic management ranging from a shared decision-making training program for antibiotic prescription in acute respiratory infection [44], management of antibiotic prescription in acute respiratory infections [43,45], inappropriate prescribing in primary care [41], a multifaceted education program to reduce antibiotic dispensing in primary care [42,48], and an antibiotic decision management guide [49].

All studies were conducted in high-income countries, except for one study that was conducted in an upper–middle-income country (China) [47]. Of the seven studies conducted in high-income countries, two were performed in the United States [41,49], two were solely performed in the United Kingdom [42,48], one was performed in six European countries (England, Wales, Belgium, the Netherlands, Spain, and Poland) [43], one was performed in Canada [44], and one was performed in the Netherlands [45]. All studies were conducted in clinical settings: either primary care or hospital/clinics. All studies received funding support from nonindustrial sponsorship, except one study where the authors received funding support from companies (Johnson & Johnson and “Research-In-Motion corporations”) [49].

For the intervention groups, three studies used blended digital education (ie, online plus traditional education such as face-to-face interactive workshops; group discussions and reflections, printed leaflets, posters) [42,44,48], three studies used online digital education, which involved online training materials as well as emails with feedback and suggestions [41,43,45]; and two studies used mobile digital education devices, namely, a mobile phone or personal digital assistant [47,49]. The duration of the interventions ranged from 1 hour [42] to 18 months [41]. Three studies did not provide information about the duration of the interventions [43,45,47].

For the control groups, one study used a traditional continuing medical education program, while the other seven studies employed usual practice (ie, participants received no additional intervention other than usual on the job training). Additionally, one study compared different forms of online digital education programs to traditional education [43].

The included studies reported outcomes such as clinical practice (n=6 studies), patient-related outcomes (n=4 studies), knowledge (n=2 studies), health care professionals’ attitude toward the intervention (n=2 studies), and economic outcomes (n=3 studies). None of the studies reported adverse or unintended effects of interventions. Table 1 presents a detailed description of the characteristics of the included studies.

**Table 1:** Characteristics of included studies.

Study author, year, study design, country	Population (n)	Setting and source of funding	Field of study	Intervention type	Duration of the intervention	Control	Outcome(s) assessed
Bochicchio, 2006, RCT^a^, US [49]	12 primary care physicians	Primary care setting, industrial funding (Johnson & Johnson, Research-In-Motion Corporation)	Antibiotic decision management guide	Mobile digital education	3 months	Traditional education (usual practice)	Knowledge
Butler, 2012, RCT, UK [48]	68 practices, 263 primary care physicians, and 480,000 patients (approximate number)	Primary care setting, nonindustrial funding	Multifaceted educational program to reduce antibiotic dispensing in primary care	Online blended education (online digital education plus traditional education)	1.5 hours	Traditional education (usual practice)	Clinical practice improvement; patient-related outcomes; economic outcome(s)
Chen, 2014, cRCT^b^, China [47]	100 practices and 479 primary care physicians	Primary care setting, nonindustrial funding	Management of upper respiratory infection	Mobile digital education	N/A^c^	Traditional education (traditional CME^d^ program)	Knowledge; clinical practice improvement; health care professionals’ attitude; economic outcome(s)
Dekker, 2018, cRCT, the Netherlands [45]	35 practices, 75 primary care physicians, and 1009 patients	Primary care setting, nonindustrial funding	Antibiotic prescription in acute respiratory infection	Online digital education	N/A	Traditional education (usual practice)	Clinical practice improvement; patient-related outcomes

Legare, 2012, cRCT, Canada [44]	9 practices, 149 primary care physicians, and 359 patients	Primary care setting, nonindustrial funding	Antibiotic prescription in acute respiratory infection	Online blended education (online digital education plus traditional education)	4 hours	Traditional education (usual practice)	Patient-related outcomes
Meeker, 2016, cRCT, USA [41]	47 practices and 248 primary care physicians	Primary care setting, nonindustrial funding	Antibiotic prescription among primary care practices	Online digital education	>18 months	Traditional education (usual practice)	Clinical practice improvement

McNulty, 2018, cRCT, UK [42]	150 practices and 166 primary care physicians	Primary care setting, nonindustrial funding	Antibiotic dispensing in primary care	Online blended education (online digital education plus traditional education)	1 hour	Traditional education (usual practice)	Clinical practice improvement

Little, 2013, cRCT, six European countries (England, Wales, Belgium, the Netherlands, Spain, and Poland) [43]	246 practices and 4264 patients	Primary care setting, nonindustrial funding	Antibiotic prescription in acute respiratory infection	Online digital education (CRP^e^ training)	N/A	Traditional education (usual practice)	Clinical practice improvement; health care professionals' attitude; patient-related outcomes; economic outcome(s)
Yardley 2013, cRCT, six European countries (as mentioned above) [39]	246 practices and 4264 patients	Primary care setting, nonindustrial funding	Antibiotic prescription in acute respiratory infection	Online digital education (enhanced-communication training)	N/A	Traditional education (usual practice)	Clinical practice improvement; health care professionals' attitude; patient-related outcomes; economic outcome(s)
Oppong 2018, cRCT, six European countries (as mentioned above) [40]	246 practices and 4264 patients	Primary care setting, nonindustrial funding	Antibiotic prescription in acute respiratory infection	Online digital education (combined CRP and enhanced-communication training)	N/A	Traditional education (usual practice)	Clinical practice improvement; health care professionals' attitude; patient-related outcomes; economic outcome(s

^a^RCT: randomized controlled trial.

^b^cRCT: cluster randomized controlled trial.

^c^N/A: not applicable.

^d^CME: continuing medical education.

^e^CRP: C-reactive protein.

### Knowledge Outcome (Postintervention)

**Figure 3:**
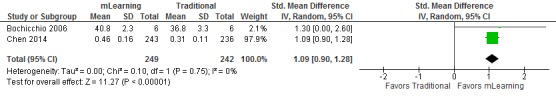
Forest plot for postintervention knowledge outcome (mobile digital education vs traditional education). mLearning: mobile learning; IV: inverse variance.

Two studies (491 participants) assessed knowledge as the primary outcome and evaluated it with multiple choice questionnaires. One study assessed short-term postintervention knowledge [47], and another study assessed both postintervention knowledge as well as knowledge retention at 3 months postintervention [49]. The pooled estimate suggests that the intervention (mobile phone or personal digital assistant device [mobile digital education]) may improve postintervention knowledge scores as compared to traditional education (SMD=1.09, 95% CI 0.90-1.28; I^2^=0%; 491 participants [2 studies], large effect size; Figure 3).

### Clinical Practice Improvement

Six studies assessed the effectiveness of online digital education (either alone or in a blended format) and mobile digital education modalities in reducing antibiotic prescribing or dispensing rate [41-43,45,47,48]. Out of six studies, four studies [41,43,45,48] favored digital education and two studies [42,47] reported no difference in the effectiveness between digital education and traditional education. Because of high heterogeneity across the studies, the results of these studies are presented narratively.

Little et al [43] assessed the effectiveness of different forms of online digital education in comparison to traditional education and reported that the combined intervention (online training on point of care C-reactive protein [CRP] test and enhanced-communication skills) was associated with the greatest reduction in prescribing rate (combined intervention: RR=0.38, 95% CI 0.25-0.55, *P*<.001; online-based CRP training: RR=0.53, 95% CI 0.36-0.74, *P*<.001; enhanced communication skills training: RR=0.68, 95% CI 0.5-0.89, *P*=.003) compared to traditional education. Meeker et al [41] assessed the use of online digital education (peer comparison via email with feedback and suggestions for improving performance) in comparison to traditional education and reported a greater reduction in antibiotic-prescribing rates in the intervention group compared to the control (difference in mean change score: –5.2%, 95% CI −6.9 to −1.6]; *P*<.001) [41]. Similarly, Dekker et al [45] reported that the antibiotic prescribing rate was lower in the online digital education group (received online learning resources) compared to the traditional education group (RR=0.65, 95% CI 0.46-0.91) [45]. Butler et al [48] reported a reduction in the total oral antibiotic dispensing rate for the study year in online blended education (4.2%, 95% CI 0.6-7.7; *P*=.02) compared to traditional education [48]. Chen et al [47] assessed the effectiveness of mobile phone text messaging in comparison to traditional education and reported no significant difference in antibiotic-prescribing rates postintervention between the groups (RR=1.02, 95% CI 0.94-1.1; *P*=.63) [47]. McNulty et al [42] compared online blended education to usual practice and found no significant difference in the antibiotic dispensing rates between the groups (dispensing rate ratio 0.973, 95% CI 0.945-1.001; *P*=.06) [42].

In general, the findings suggest that, compared to traditional education, a variety of different forms of online digital education (eg, email with feedback and suggestions, studying online learning resources, and online training on point-of-care CRP test) may improve primary care physicians’ clinical practice and reduce antibiotic prescriptions or dispensing rates. The combined training of online-based point-of-care CRP test and communication skills was found to be more effective than other forms of online digital education and traditional education in reducing the antibiotic-prescribing rate among primary care practice.

### Primary Care Physicians’ Attitude Toward the Intervention

Two studies assessed postintervention attitudes toward different forms of digital education on the topic of antibiotic prescriptions. Chen et al [47] assessed only the attitude of primary care physicians in the intervention group toward the intervention (ie, text messages containing evidence-based recommendations). One-third of the participants in the intervention group reported that they frequently adopted the recommendations in their clinical decision making, and 95% wanted to continue receiving the text messages. Little et al [43] compared three different forms of online training on antibiotic prescription and assessed primary care physicians’ attitudes towards the intervention. The study reported no difference between the participants of different online training groups regarding their perceptions of any potential damage that the intervention could have had on their relationships with patients and their confidence in reducing prescription Multimedia Appendix 4) [43].

### Patient-Related Outcomes

Four studies assessed diverse patient-related outcomes [43-45,48] and reported mixed or inconclusive findings. Butler et al [48] reported there was no difference in the postintervention hospital admission rate (–1.9% reduction in the intervention group, 95% CI –13.2 to 8.2; *P*=.72) and reconsultation rate in the 7-day postintervention median scores (MD=–0.65, 95% CI –1.69 to 0.55; *P*=.45) between the intervention (ie, online blended digital education) and the traditional education (ie, usual practice) groups.

Dekker et al [45] reported that the reconsultation rate for children within the same disease episode was lower in the intervention group receiving online digital education than the group receiving traditional education (RR=0.66, 95% CI 0.38-1.16). However, the same study reported that the probability of consultation for a new respiratory tract infection within 6 months (RR=1.06, 95% CI 0.72-1.58) and hospital referral (RR=0.66, 95% CI 0.31-1.40) did not differ significantly.

Legare et al [44] reported that an online-based shared decision-making program enhanced patient participation in decision making and led to fewer patients deciding to use antibiotics for acute respiratory infections compared to traditional education (RR=0.48, 95% CI 0.34-0.68). However, there was no difference in the effects between the two groups regarding other patient-related outcomes such as adherence to the decision (SMD=–0.82, 95% CI –2.23 to 0.59), repeated consultations (SMD=0.80, 95% CI –0.60 to 2.20), decisional regret (SMD=0.25, 95% CI –1.07 to 1.57), quality of life (SMD=0.04, 95% CI –1.27 to 1.36), and intention to engage in shared decision making in future consultations regarding the use of antibiotics for acute respiratory infections (SMD= 0.16, 95% CI –1.16 to 1.47).

Little et al [43] reported that compared to usual practice, online digital education about CRP training had little or no difference in postintervention patient-related outcomes such as patient enablement (SMD=–0.11, 95% CI –0.24 to 0.01), patients’ satisfaction with consultation (SMD=–0.09, 95% CI –0.22 to 0.03), and antibiotic usage (SMD=0.13, 95% CI 0.01-0.26). Similarly, the study reported no difference in postintervention patient outcomes with the use of online training on enhanced communication skills compared to traditional education (Multimedia Appendix 4).

### Economic Outcomes

Three studies reported that the intervention costs were lower than those of traditional education [43,47,48]. Butler et al [48] assessed the effects of online blended education compared to traditional education and reported that the mean cost of the program was £2923 (~US $4559) per practice (SD £1187 [~US $1852]), and there was a 5.5% reduction in the cost of dispensed antibiotics in the intervention group compared with the control group (–0.4% to 11.4%), which was equivalent to a reduction of about £830 (~US $1295) a year for an average intervention practice. Chen et al [47] also reported that total expenditure on text messages for each health worker in the intervention group was less than 2 ¥ (US $0.32) and for the control group with traditional education, it cost 560 ¥ (US $89.96) per health worker, for printed materials, accommodation, and transportation costs. This amounts to a 280-fold difference per person. Little et al [43] reported that online-based communication skills training was more cost-effective than traditional education (10% cost reduction) if the cost of antibiotic resistance was accounted for (€83.21 [~US $110.67] vs €92.46 [US $122.97]).

None of the included studies reported health care professionals’ post-intervention skills, satisfaction, and adverse or untoward effects of digital education.

**Figure 4:**
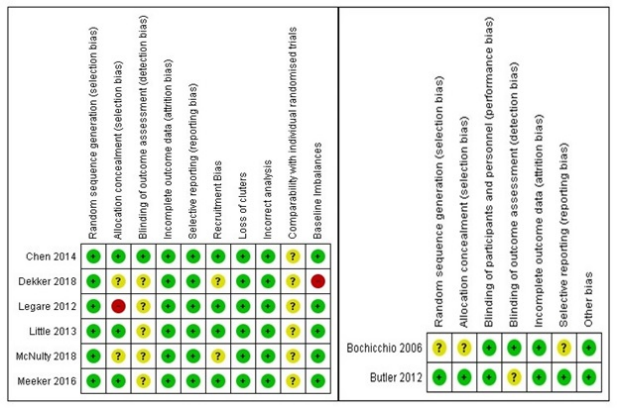
Risk of bias summary. Green: high risk of bias; red: low risk of bias; yellow: unclear risk of bias.

The overall risk of bias was high or unclear for most of the studies. We judged high or unclear risk of bias for six studies where risk of bias was mainly unclear in selection, detection, recruitment, reporting, and comparability with individual RCT (for cRCTs) domains due to unclear/lack of information (in four studies), and high risk of allocation concealment and other bias due to baseline differences (in two studies). In two studies, we judged the risk of bias as low, as the studies provided detailed information on random sequence generation, allocation concealment, blinding of outcome assessment, attrition rate, reporting of outcomes of interest, and baseline differences (Figure 4).

## Discussion

### Principal Findings

Our review evaluated the effectiveness of digital education for health professions on antibiotic management compared with traditional education. Our results suggest that the use of online and mobile digital education in the management of antibiotics for postregistration health care professionals is associated with increased professional knowledge compared with traditional education. Health care professionals’ clinical practice improvement (reduction in antibiotics prescribing or dispensing) was evaluated in six of eight included studies, which showed that online digital education may be more effective than traditional education. We found that certain types of mobile digital education modes were associated with significantly lower costs compared to traditional education. Overall, the risk of bias was high or unclear due to a lack of information mainly in relation to selection, detection, recruitment, reporting, and comparability with individual RCT (for cRCTs) domains.

Literature has shown that primary care settings are the most significant contributor to avenues for inappropriate antibiotic prescribing. There is therefore an emphasis on interventions promoting prudent prescribing in primary care, as detailed globally in national action plans on AMR [50]. Correspondingly, studies included in our review had a primary care focus—to compare the effectiveness of different educational approaches such as nondigital interventions (ie, delayed prescriptions or face-to-face interactive sessions) with digital education (ie, delivered via mobile digital education or online digital education) to improve antibiotic management in these settings [39-49].

Past reviews [29,30] highlighted that antibiotic management can be improved by educational interventions. However, these reviews focused on the effectiveness of nondigital education (ie, traditional education such as the use of text-based or paper-based interventions and quality improvement strategies). To the best of our knowledge, no reviews evaluated the effectiveness of digital education on the management of antibiotics for health care professionals. Our review provides the most comprehensive and up-to-date evaluation of digital education on antibiotic prescription in health care professionals. It shows the potential effectiveness for the use of certain types of digital technologies.

### Strengths and Limitations

Our review has a number of strengths. We used robust methodology to synthesize the evidence by following gold-standard Cochrane guidelines while conducting this systematic review. We used a comprehensive search strategy and searched all major bibliographic databases for eligible studies.

However, our review has some limitations, which may affect the generalizability of the evaluated evidence. First, all included studies were conducted in primary care settings, and all participants were primary care physicians. Therefore, there is a need for studies focusing on other types of health care professionals such as dentists and allied health care professionals and on other settings such as pharmacies and other hospital practices. Antibiotic stewardship activity is increasingly focusing on the role of the community pharmacist in antibiotic prescription [51]. Second, all included studies focused on mobile digital education and online digital education. The effectiveness of other digital education modalities such as virtual reality, virtual patient scenarios, and offline digital education are underrepresented in the literature. Third, there is a lack of data reported in the included studies on health care professionals' postintervention skills, attitude, satisfaction, and economic and patient-related outcomes. None of the included studies evaluated untoward or adverse effects of the intervention for patients (eg, patient mortality, morbidity, and medical errors) and for learners (eg, dizziness, vertigo, and loss of attention). Due to limited data from the included studies and heterogeneity across the studies, we could not perform any sensitivity analysis or subgroup analyses that we had initially planned. Moreover, we were unable to evaluate some of the risk of bias domains due to poor description of the studies and lack of information within the study, which may affect the comprehensiveness of the risk of bias assessment. We analyzed postintervention data for two studies that reported knowledge outcome, as we could not calculate mean change score for the comparisons. Fourth, most of the included studies (7/8) were from high-income countries and no or limited data were from low- and middle-income countries, indicating that the depth of evidence related to any given outcome is mostly limited to high-income countries. Fifth, our review focuses only on the effectiveness of the digital education for antibiotic management training and does not address other important aspects such as potential barriers and facilitators or attrition rates. Lastly, only three studies assessed the cost of intervention and none reported on health care professionals’ postintervention skills or adverse/untoward effects of digital education, thereby limiting the overall completeness and applicability of evidence.

### Future Research

While the evidence included in our review is limited, it shows the potential effectiveness and applicability of certain digital education modalities such as mobile learning and online digital education in postregistration health professions education for antibiotic management. Future research should explore the effectiveness of other technologies such as virtual reality, virtual patient simulations, or serious gaming for training of health care professionals in diverse settings and measure outcomes such as change in skills and satisfaction.

### Conclusions

Our findings suggest the potential effectiveness of mobile learning and online digital education for health professions education in antibiotic management. There is a lack of studies evaluating the effectiveness in low- and middle-income countries and of other forms of digital education such as virtual reality, virtual patient education, and offline digital education on antibiotic management. Future research should focus on assessing the effectiveness of different digital education modalities on health care professionals’ antibiotic management across different settings. This should also be extended to the role of digital education in the management of antimicrobials, in general. There is also a need to integrate the available evidence into health professions education programs or clinical practice, especially on the use of online and mobile digital education modalities for postregistration health professions education on antibiotic management.

## Appendix

Multimedia Appendix 1 Description of the digital education modalities.

Multimedia Appendix 2 MEDLINE (OVID) search strategy.

Multimedia Appendix 3 Lists of clinical trial registries.

Multimedia Appendix 4 Results of included studies.

## Abbreviations

AMR: antimicrobial resistance
CME: continuing medical education
cRCT: cluster randomized controlled trial
CRP: C-reactive protein
PROSPERO: International Prospective Register of Systematic Reviews
RCT: randomized controlled trial
RR: risk ratio
SMD: standardized mean difference
WHO: World Health Organization
