# Supplementary material for: Health Professions Digital Education on Antibiotic Management: Systematic Review and Meta-Analysis by the Digital Health Education Collaboration
Source: J Med Internet Res. 2019 Sep 12;21(9):e14984. doi: 10.2196/14984 (PMC6746065; doi:10.2196/14984)
Supplement: Multimedia Appendix 2 [file jmir_v21i9e14984_app2.pdf]

Multimedia Appendix 2. MEDLINE (OVID) Search Strategy

1. exp education, professional/ not education, veterinary/
2. Education, Predental/
3. Education, Premedical/
4. exp Students, Health Occupations/
5. ((medic* or premedic* or dent* or laborator* or predent* or midwi?e* or nurs* or nutrition* or orthop* or podiat* or pharmac* or psycholog* or psychiatr* or health or healthcare or occupational therap* or physiotherap* or physical therap* or clinical or surg* or radiolog* or obstetric* or gyn?ecolog* or orthodont* or An?esthesi* or Dermatolog* or Oncolog* or Rheumatolog* or Neurolog* or Patholog* or P?ediatric* or Cardiolog* or Urolog*) adj3 (student* or graduate* or undergraduate* or staff or personnel or practitioner* or clerk* or fellow* or internship* or residen* or educat* or train* or novice* or tutor*)).tw,kf.
6. or/1-5
7. Computer-Assisted Instruction/
8. exp Internet/
9. Computer Simulation/
10. Patient Simulation/
11. software/
12. Mobile Applications/
13. User-Computer Interface/
14. Video Games/
15. Web Browser/
16. Education, Distance/
17. Computers/
18. exp Microcomputers/
19. exp Cell Phones/
20. Games, Experimental/
21. exp Models, Anatomic/
22. Audiovisual Aids/
23. Educational Technology/
24. Electronic Mail/
25. exp Telemedicine/
26. Telenursing/
27. Telecommunications/
28. Webcasts/
29. exp Videoconferencing/
30. ((computer* or digital* or hybrid or blended or mixed mode or distance or remote* or electronic or mobile or online* or interactiv* or multimedia or internet or web* or virtual* or game* or gaming or Videogame* or Videogaming) adj3 (classroom* or course* or educat* or instruct* or learn* or lecture* or simulat* or train* or teach* or tutor* or platform*)).tw,kf.
31. (Simulat* adj3 (course* or educat* or instruct* or learn* or train* or teach* or platform* or high-fidelity)).tw,kf.
32. e-learn*.tw,kf.
33. elearn*.tw,kf.

34. m-learn*.tw,kf.
35. mlearn*.tw,kf.
36. smartphone*.tw,kf.
37. smart-phone*.tw,kf.
38. ((mobile or cell) adj2 phone*).tw,kf.
39. iphone*.tw,kf.
40. android*.tw,kf.
41. ipad*.tw,kf.
42. Personal digital assistant*.tw,kf.
43. handheld computer*.tw,kf.
44. Mobile App?.tw,kf.
45. Mobile Application?.tw,kf.
46. webcast*.tw,kf.
47. webinar*.tw,kf.
48. flipped classroom*.tw,kf.
49. Serious game*.tw,kf.
50. Serious gaming.tw,kf.
51. Patient Simulat*.tw,kf.
52. Virtual patient*.tw,kf.
53. ((educat* or instruct* or learn* or simulat* or train* or teach* or interactiv*) adj2 technolog*).tw,kf.
54. Massive Open Online Course?.tw,kf.
55. Mooc?.tw,kf.
56. (Canvas network or Coursera or Coursesites or edx or Futurelearn or iversity or miriada x or moodle or novoed or openlearning or open2study or plato or spoc or udacity or pingpong).tw,kf.
57. or/7-56
58. 6 and 57
59. Education.fs.
60. Education/
61. Teaching/
62. Learning/
63. exp Inservice Training/
64. Curriculum/
65. educat*.tw,kf.
66. learn*.tw,kf.
67. train*.tw,kf.
68. instruct*.tw,kf.
69. teach*.tw,kf.
70. or/59-69
71. Health Personnel/
72. exp Allied Health Personnel/
73. Anatomists/
74. "Coroners and Medical Examiners"/
75. exp Dental Staff/
76. exp Dentists/

77. Health Educators/
78. Infection Control Practitioners/
79. Medical Laboratory Personnel/
80. exp Medical Staff/
81. exp Nurses/
82. exp Nursing Staff/
83. Personnel, Hospital/
84. Pharmacists/
85. exp Physicians/
86. Physician*.tw,kf.
87. Doctor*.tw,kf.
88. Nurs*.tw,kf.
89. Surg*.tw,kf.
90. Health Personnel.tw,kf.
91. healthcare professional*.tw,kf.
92. radiolog*.tw,kf.
93. dentist*.tw,kf.
94. Pharmacist*.tw,kf.
95. Hospital Administrator*.tw,kf.
96. Podiatr*.tw,kf.
97. Psycholog*.tw,kf.
98. Psychiatr*.tw,kf.
99. An?esthesi*.tw,kf.
100. Clinician*.tw,kf.
101. Dermatolog*.tw,kf.
102. General practioner*.tw,kf.
103. Cardiolog*.tw,kf.
104. Oncolog*.tw,kf.
105. Rheumatolog*.tw,kf.
106. Neurolog*.tw,kf.
107. Patholog*.tw,kf.
108. P?ediatric*.tw,kf.
109. Physiotherap*.tw,kf.
110. Physical therap*.tw,kf.
111. Occupational therap*.tw,kf.
112. dieti?ian*.tw,kf.
113. Dietetic*.tw,kf.
114. midwi?e*.tw,kf.
115. nutrition*.tw,kf.
116. orthopti*.tw,kf.
117. obstetric*.tw,kf.
118. gyn?ecolog*.tw,kf.
119. orthodont*.tw,kf.
120. Urolog*.tw,kf.
121. or/71-120
122. Health Occupations/

123. exp Allied Health Occupations/
124. Biomedical Engineering/
125. Chiropractic/
126. exp Dentistry/
127. exp Evidence-Based Practice/
128. exp Medicine/
129. exp Nursing/
130. Dietetics/
131. Optometry/
132. Orthoptics/
133. exp Pharmacology/
134. exp Pharmacy/
135. Podiatry/
136. Psychology, Medical/
137. Serology/
138. Specialization/
139. exp Surgical Procedures, Operative/
140. exp Radiography/
141. or/122-140
142. 121 or 141
143. 57 and 70 and 142
144. Psychomotor Performance/
145. motor skills/
146. ((psychomotor or procedural or technical) adj3 skill*).tw,kf.
147. (psychomotor adj3 performance).tw,kf.
148. or/144-147
149. 6 and 148
150. 58 or 143 or 149
151. limit 150 to yr="1990 -Current"
152. randomized controlled trial.pt.
153. controlled clinical trial.pt.
154. randomized.ti,ab.
155. placebo.ti,ab.
156. drug therapy.fs.
157. randomly.ti,ab.
158. trial.ti,ab.
159. groups.ti,ab.
