# Supplementary material for: Health Professions Digital Education on Antibiotic Management: Systematic Review and Meta-Analysis by the Digital Health Education Collaboration
Source: J Med Internet Res. 2019 Sep 12;21(9):e14984. doi: 10.2196/14984 (PMC6746065; doi:10.2196/14984)
Supplement: Multimedia Appendix 3 [file jmir_v21i9e14984_app3.pdf]

Multimedia Appendix 3: Search strategy for International Clinical Trials Platform (ICTRP) – WHO

Source Link: http://apps.who.int/trialsearch/AdvSearch.aspx

Data sets from data providers are updated every Wednesday evening according to the following schedule:

Every week:

- Australian New Zealand Clinical Trials Registry, last data file imported on **10 September 2018**
- Chinese Clinical Trial Registry, last data file imported on **10 September 2018**
- ClinicalTrials.gov, last data file imported on **10 September 2018**
- EU Clinical Trials Register (EU-CTR), last data file imported on **10 September 2018**
- ISRCTN, last data file imported on **10 September 2018**
- The Netherlands National Trial Register, last data file imported on **10 September 2018**

Every 4 weeks:

- Brazilian Clinical Trials Registry (ReBec), last data file imported on **10 September 2018**
- Clinical Trials Registry - India, last data file imported on **11 September 2018**
- Clinical Research Information Service - Republic of Korea, last data file imported on **11 September 2018**
- Cuban Public Registry of Clinical Trials, last data file imported on **27 August 2018**
- German Clinical Trials Register, last data file imported on **27 August 2018**
- Iranian Registry of Clinical Trials, last data file imported on **11 September 2018**
- Japan Primary Registries Network, last data file imported on **10 September 2018**
- Pan African Clinical Trial Registry, last data file imported on **10 September 2018**

- Sri Lanka Clinical Trials Registry, last data file imported on **10 September 2018**
- Thai Clinical Trials Register (TCTR), last data file imported on **10 September 2018**
- **New** Peruvian Clinical Trials Registry (REPEC), last data file imported on **10 September 2018**

SEARCH 1

Using the title field in the advanced search, searching ALL registered trials

e-learning OR elearning OR m-learning OR mlearning OR "Canvas network" OR Coursera OR Coursesites OR edx OR Futurelearn OR iversity OR "miriada x" OR moodle OR novoed OR openlearning OR open2study OR plato OR spoc OR udacity OR pingpong OR "Massive Open Online Course*" OR Mooc OR Moocs OR smartphone* OR smart-phone* OR iphone* OR android* OR ipad* OR "personal digital assistant*" OR "handheld computer*" OR "mobile app" OR "mobile apps" OR "mobile application" OR "mobile applications" OR webcast* OR webinar* OR "flipped classroom*" OR "serious game*" OR "serious gaming" OR "patient simulat*" OR "virtual patient*" OR "psychomotor performance"

SEARCH 2

Using the title field in the advanced search, searching ALL registered trials

(comput* OR digital* OR hybrid OR blended OR "mixed mode" OR distance OR remote* OR electronic OR mobile OR online* OR interactiv* OR multimedia OR internet OR web* OR virtual OR game* OR gaming OR videogame* OR videogaming OR simulat* OR virtual OR technolog*)

AND

(classroom* OR course* OR educat* OR instruct* OR learn* OR lecture* OR simulat* OR train* OR teach* OR tutor* OR platform* OR "high-fidelity")
