# Supplementary material for: Health Professions Digital Education on Antibiotic Management: Systematic Review and Meta-Analysis by the Digital Health Education Collaboration
Source: J Med Internet Res. 2019 Sep 12;21(9):e14984. doi: 10.2196/14984 (PMC6746065; doi:10.2196/14984)
Supplement: Multimedia Appendix 4 [file jmir_v21i9e14984_app4.pdf]

Multimedia Appendix 4: Results of the included studies									
**Study ID (Author Year), Study Design, Country**	**Intervention Type**	**Population (N)**	**Field of Study**	**Knowledge**	**Clinical practice improvement**	**Healthcare Professionals' Attitude**	**Patient-related outcomes**	**Economic outcome(s)**	**Conclusion**
Bochicchio 2006, RCT, USA	I: Mobile digital education<br><br>C: Traditional education (Usual practice)	12 primary care physicians	Antibiotic decision management guide	Post-intervention mean score on 50 items MCQ, SMD=1.3 [0.00, 2.6]	N/A	N/A	N/A	N/A	**Knowledge:** Mobile digital education > Traditional education
Butler 2012, RCT, UK	I: Online blended education (Online digital education plus traditional education)<br><br>C: Traditional education (Usual practice)	68 practices, 263 primary care physicians and 480,000 patients (approximate number)	Multifaceted educational program to reduce antibiotic dispensing in primary care	N/A	Reported a reduction in the rate of total oral antibiotic dispensing for the study year in online blended education (4.2%, 95% CI 0.6% to 7.7%, P=0.02)	N/A	No difference in post-intervention hospital admission rate (MD=-1.9%, 95% CI: -13.2 to 8.2, P=0.72) and re-consultation rate at seven day post-intervention median scores (MD=-0.65 (95% CI -1.69 to 0.55, P=0.446) between the intervention (i.e. online blended digital education) and the traditional education groups (i.e. usual practice).	Mean cost of the programme (intervention) was £2923 per practice (SD £1187). There was a 5.5% (-0.4% to 11.4%) reduction in the cost of dispensed antibiotics in the intervention group compared with the control group which was equivalent to a reduction of about £830 a year for an average intervention practice.	**Clinical practice improvement:** Online blended education > Traditional education<br><br>**Patient-related outcomes:** Online blended education = Traditional education<br><br>**Economic outcome(s):** Online blended education > Traditional education

Chen 2014, cRCT, China	I: Mobile digital education<br><br>C: Traditional education (Traditional CME program)	100 practices and 479 primary care physicians	Management of upper respiratory infection	Post-intervention mean score on 10 items MCQ, SMD=1.09 [0.96, 1.23]	Antibiotic prescribing rate for intervention group in comparison to the control (RR=1.02, 95% CI: 0.94 to 1.1, P=0.63).	One-third of the participants in the intervention group reported that they frequently adopted the recommendations in their clinical decision-making and 95% wanted to continue receiving the text messages.	N/A	Total cost in the intervention group = <2 Yuan (US $0.32) per health worker (i.e. cost on text messages)<br><br>Total cost in traditional education group = 560 Yuan (US $89.96) per health worker (for printed materials, accommodation and transportation costs)	**Knowledge:** Mobile digital education > Traditional education<br><br>**Clinical practice improvement:** Mobile digital education = Traditional education<br><br>**Healthcare Professionals' Attitude:** (no comparative data)<br><br>**Economic outcome(s):** Mobile digital education > Traditional education
Dekker 2018, cRCT, the Netherlands	I: Online digital education<br><br>C: Traditional education (Usual practice)	35 practices, 75 primary care physicians and 1009 patients	Antibiotic prescription in acute respiratory infection	N/A	Antibiotic prescribing rate was lower in the online digital education compared to traditional education: RR=0.65 (95% CI 0.46-0.91)	N/A	Re-consultation rate for children within the same disease episode was lower in the intervention group receiving online digital education compared to traditional education (RR=0.66, 95% CI 0.38 to 1.16).	N/A	**Clinical practice improvement:** Online digital education > traditional education (usual practice)<br><br>**Patient-related outcomes:** Online digital education = traditional education (usual practice)
Legare 2012, cRCT, Canada<br><br>Couet 2014,	I: Online blended education (Online digital education plus	9 practices, 149 primary care physicians and 359 patients	Antibiotic prescription in acute respiratory infection	N/A	N/A	N/A	Patients deciding to use antibiotics for acute respiratory infections compared to traditional education	N/A	**Patient-related outcomes:** Online blended education = Traditional education

cRCT, Canada	traditional education)<br><br>C: Traditional education (Usual practice)						(RR=0.48, 95% CI 0.34 to 0.68).<br><br>Decisional regret: SMD=0.25 (95% CI -1.07, 1.57)<br><br>Intention to engage in shared decision-making: SMD= 0.16 (95% CI -1.16, 1.47)<br><br>Quality of life*: SMD=0.04 (95% CI -1.27, 1.36)<br><br>Adherence to the decision: SMD=-0.82 (95% CI -2.23, 0.59)<br><br>Repeated consultations: SMD=0.80 (95% CI -0.60, 2.20)		
Meeker 2016, cRCT, USA	I: Online digital education<br><br>C: Traditional education (Usual practice)	47 practices and 248 primary care physicians	Antibiotic prescription among primary care practices	N/A	Reduction in antibiotic prescribing rates in the intervention group compared to traditional education (Difference in mean change score: -5.2% [95%CI, −6.9%to −1.6%]; P<0.001).	N/A	N/A	N/A	**Clinical practice improvement:** Online digital education > traditional education
McNulty 2018, cRCT, UK	I: Online blended education	150 practices and 166	Antibiotic dispensing in	N/A	Antibiotics Dispensing rate ratio	N/A	N/A	N/A	**Clinical practice improvement:**

	(Online digital education plus traditional education)<br><br>C: Traditional education (Usual practice)	primary care physicians	primary care		0.973, 95% CI 0.945 to 1.001, P=0.06)				Online blended education = traditional education (reduction in antibiotic dispensing)
Little 2013, cRCT, six European countries (England, Wales, Belgium, the Netherlands, Spain and Poland)<br><br>Yardley 2013, cRCT, six European countries<br><br>Oppong 2018, cRCT, six European countries	Ia: Online digital education (CRP training)<br><br>Ib: Online digital education (enhanced-communication training)<br><br>Ic: Online digital education (combined training of CRP and enhanced-communication)<br><br>C: Traditional education (Usual practice)	246 practices and 4264 patients	Antibiotic prescription in acute respiratory infection	N/A	Antibiotic prescribing rate was lower with CRP training compared to traditional education: RR=0.53, 95% CI 0.36 to 0.74, P < 0.0001<br><br>With enhanced-communication training compared to traditional education: RR=0.68, 95% CI 0.5 to 0.89, P=0.003<br><br>Combined training compared to traditional education: RR=0.38, 95% CI 0.25 to 0.55, P < 0.0001	N/A	Patient enablement:<br><br>CRP vs traditional education: SMD=-0.11 (95% CI -0.24 to 0.01)<br><br>Enhanced communication vs. traditional education: SMD=0.07 (95% CI -0.05 to 0.19)<br><br>Combined group vs. traditional education: SMD= -0.01 (95% CI -0.13 to 0.11)<br><br>Satisfaction with consultation:<br><br>CRP vs. traditional education: SMD=-0.09 (95% CI -0.22 to 0.03)<br><br>Enhanced communication vs. education:	10% cost reduction with online training compared to traditional education if the cost of antibiotic resistance was accounted for [€ 83.21 vs € 92.46].	**Clinical practice improvement:**<br><br>Online digital education > traditional education<br><br>**Patient-related outcomes:**<br>Online digital educations ≥ traditional education (usual care)<br><br>**Economic outcome(s):**<br>Online based communication skills training was more cost-effective than traditional education

							SMD=0.12 (95% CI -0.01 to 0.24)<br><br>Combined group vs. traditional education: SMD= 0.05 (95% CI -0.08 to 0.17)<br><br><u>Taking antibiotics can do more harm than good:</u><br><br>CRP vs. traditional education: SMD=0.13 (95% CI 0.01 to 0.26)<br><br>Enhanced communication vs. traditional education: SMD=0.11 (95% CI -0.01 to 0.24)<br><br>Combined group vs. traditional education: SMD= 0.14 (95% CI 0.02 to 0.26)		

*Pooled estimate on physical and mental scales.
Abbreviations: I=Intervention, C=Control group, CRP= C Reactive Protein, N/A=Not Available, RCT=Randomized Control Trial, cRCT=cluster RCT, SMD=Standarized Mean Difference, CI=Confidence Interval, RR=Risk Ratio, CME=Continuing Medical Education, MCQ=Multiple Choice Question, UK=United Kingdom, USA=United States of America
